# MHC class I expression in intestinal cells is reduced by rotavirus infection and increased in bystander cells lacking rotavirus antigen

**DOI:** 10.1038/s41598-017-18464-x

**Published:** 2018-01-08

**Authors:** Gavan Holloway, Fiona E. Fleming, Barbara S. Coulson

**Affiliations:** Department of Microbiology and Immunology, The University of Melbourne at the Peter Doherty Institute for Infection and Immunity, Melbourne, Victoria, Australia

## Abstract

Detection of viral infection by host cells leads to secretion of type I interferon, which induces antiviral gene expression. The class I major histocompatibility complex (MHCI) is required for viral antigen presentation and subsequent infected cell killing by cytotoxic T lymphocytes. STAT1 activation by interferon can induce NLRC5 expression, promoting MHCI expression. Rotavirus, an important pathogen, blocks interferon signalling through inhibition of STAT1 nuclear translocation. We assessed MHCI expression in HT-29 intestinal epithelial cells following rotavirus infection. MHCI levels were upregulated in a partially type I interferon-dependent manner in bystander cells lacking rotavirus antigen, but not in infected cells. MHCI and NLRC5 mRNA expression also was elevated in bystander, but not infected, cells, suggesting a transcriptional block in infected cells. STAT1 was activated in bystander and infected cells, but showed nuclear localisation in bystander cells only. Overall, the lack of MHCI upregulation in rotavirus-infected cells may be at least partially due to rotavirus blockade of interferon-induced STAT1 nuclear translocation. The reduced MHCI protein levels in infected cells support the existence of an additional, non-transcriptional mechanism that reduces MHCI expression. It is possible that rotavirus also may suppress MHCI expression *in vivo*, which might limit T cell-mediated killing of rotavirus-infected enterocytes.

## Introduction

The class I major histocompatibility complex (MHCI) is ubiquitously expressed on the cell surface and required for recognition and subsequent killing of infected cells by cytotoxic T lymphocytes (CTLs). Following virus infection, cytoplasmic viral proteins are processed in the proteasome and transported as peptides into the ER lumen by the transporter associated with antigen presentation proteins, TAP1 and TAP2^1^. Here, peptide antigens are loaded onto MHCI molecules, which follow a secretory path from the ER to the cell surface, where they present their associated peptide antigens extracellularly. Upon recognition of peptide/MHCI by antigen-specific CTLs, apoptosis is induced in the target cell.

MHCI expression is controlled by several mechanisms. The HLA-A and HLA-B genes, encoding two of the three major human MHCI types, are regulated by proximal promoters containing NF-κB-binding elements, an interferon (IFN)-stimulated response element (ISRE), as well as an additional region that binds nucleotide-binding domain and leucine-rich repeat containing protein CARD domain-containing 5 (NLRC5) and several other transcription factors in what has been termed the NLRC5 enhanceosome^2^. *NLRC5* itself is regulated by an IFN-γ-activated sequence (GAS) that binds STAT1 homodimers, and also an ISRE that binds the IFN response factor (IRF) 1. *IRF1* contains a GAS element in its promoter. Therefore, activation of STAT1 by IFN-γ or type I IFN (IFN-α/β) can induce IRF1 and NLRC5 expression, which in turn promote MHCI expression^2^. Cytokines that activate NF-κB, such as TNF, can also positively regulate MHCI. Other genes required for peptide presentation on MHCI, including TAP1/2, LMP2 and β2-microglobulin, have upstream sequences similar to the NLRC5 enhanceosome-binding elements of HLA-A and HLA-B, so are co-ordinately regulated.

Rotavirus, a non-enveloped dsRNA virus of the *Reoviridae* family, is the leading etiologic agent of severe infantile gastroenteritis. Control of rotavirus replication and clearance in the host involves both innate and adaptive immune responses^3,4^. Innate responses to rotavirus require intact IFN-α/β- and IFN-λ-dependent signalling and are initiated by RIG-I, MDA5 and TLR7^3,5–8^. Rotavirus has evolved several mechanisms to evade the innate immune system including the non-structural protein 1 (NSP1)-mediated degradation of IRF3, IRF5, IRF7 and IRF9 as well as β-TrCP, a protein required for NF-κB activation^9–13^. In addition, rotavirus interferes with the antiviral protein RNase L through the action of the viral protein (VP) 3^14^. Rotavirus also inhibits IFN signaling in infected cells by blocking the nuclear translocation of STAT1 and STAT2^15,16^.

Due to the importance of MHCI in CTL recognition of virus-infected cells and the ability of rotavirus to inhibit STAT1 signaling (a process intimately linked to MHCI regulation), we assessed MHCI expression in an intestinal cell culture model following rotavirus infection. It was found that total MHCI was upregulated in bystander cells lacking rotavirus antigen, but not in infected cells, and that MHCI upregulation was at least partially dependent upon type I IFN signalling. MHCI and NLRC5 mRNA expression was elevated in bystander, but not infected cells, supporting the possibility of a transcriptional block as a mechanism for the lack of MHCI elevation in infected cells. In addition, MHCI levels in infected cells were reduced compared to mock-infected cells, suggesting an additional non-transcriptional mechanism of MHCI downregulation. These findings provide preliminary evidence to support the hypothesis that inhibition of MHCI expression may be important for immune evasion by rotavirus.

## Results

### Rotavirus downregulates MHCI expression in infected intestinal epithelial cells but upregulates MHCI in bystander uninfected cells

**Figure 1:**
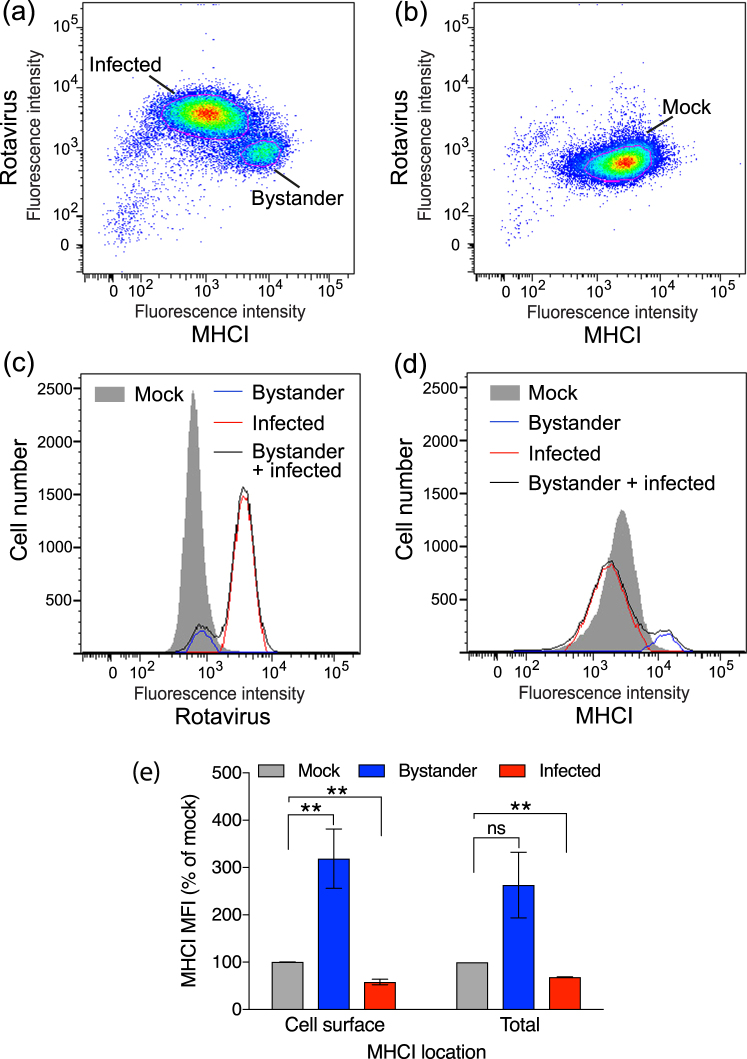
Levels of cell-surface and total MHCI following rotavirus infection of HT-29 cells. Cells were mock-infected or infected with RRV at a multiplicity of infection (m.o.i.) of 1. After 16 h cells were fixed, stained with antibodies to MHCI (Surface) then permeabilized and stained with antibodies to rotavirus and analysed by flow cytometry. Representative dot plots of rotavirus-exposed cells with the Bystander and Infected gates indicated with a red line (**a**) and mock-infected cells with the Mock gate indicated similarly (**b**) are shown. The fluorescence intensity dot-plots were set so the mock-infected cell population fell centrally on the plot, to facilitate detection of both increased and decreased intensities in virus-exposed cells. (**c**) Representative histograms of rotavirus antigen levels on the combined (Bystander + infected) and gated (Bystander, Infected) populations of rotavirus-exposed cells, and the gated mock-infected cells (Mock). (**d**) The histograms of MHCI levels on the same cell populations analysed for (**c**). In (**e**), cells were infected and stained for cell surface MHCI as above (Cell surface), or fixed and permeabilized, stained for MHCI and rotavirus (Total), and analysed by flow cytometry. The mean ± S.D. of the geometric mean fluorescence intensity (MFI) of MHCI (percentage of mock-infected cells) from three (Cell surface) or two (Total) independent experiments is shown. **P < 0.05; ns P > 0.05.

We determined cell-surface MHCI (HLA-A/B/C) and intracellular rotavirus antigen levels by flow cytometry in HT-29 cell cultures inoculated with the Rhesus monkey rotavirus strain RRV, and in mock-infected HT-29 cells. At 16 h post-exposure to RRV at a m.o.i. of 1, dot plot analysis revealed two distinct cell populations (Fig. 1a). The smaller population (~10% of cells) showed a similar (background) level of rotavirus staining to mock-infected cells, but exhibited elevated surface MHCI levels over mock-infected cells (Fig. 1a,b). This smaller population is referred to here as bystander cells, as these cells showed undetectable rotavirus antigen levels and thus did not support productive virus replication. The larger population (~90% of cells) showed fluorescence shifts indicative of positive rotavirus staining and reduced MHCI levels.

Elliptical gates were applied to most appropriately capture each of the two overwhelmingly dominant cell populations in the dot plots, and the levels of rotavirus antigen (Fig. 1c) and MHCI (Fig. 1d) were calculated and displayed as histograms. For the RRV-exposed cell cultures, the cell population prior to application of these elliptical gates, containing both the infected and bystander cells, also was analysed for comparison. These analyses confirmed that the population of cells expressing elevated MHCI contained undetectable virus antigen levels, as the histogram of these cells sat under that of the mock-infected cells. These studies also substantiated the reduced MHCI levels expressed by the infected cell population.

MHCI levels on infected and bystander cells were analysed as above from multiple independent experiments. Compared to mock-infected cells, cells expressing rotavirus antigen showed a mean decrease in surface MHCI level of 42% (p < 0.05; Fig. 1e). In contrast, rotavirus antigen-negative (uninfected) bystander cells showed a 320% increase in surface MHCI expression compared to mock-infected cells (p < 0.05; Fig. 1e). A trend towards a similar increase in total MHCI expression in bystander cells (p > 0.05), and a decrease in total MHCI in infected cells (p < 0.05) that was similar to the decrease in surface MHCI expression, were observed using cells that were permeabilized before staining for MHCI and rotavirus antigen (Fig. 1e). This shows that surface and probably total MHCI expression in an intestinal cell culture system is upregulated in bystander cells but downregulated in rotavirus-infected cells.

In contrast to the above results, a past study conducted in our laboratory detected no changes in MHCI levels in RRV-infected and bystander HT-29 cells, under similar conditions and analysis protocols to those of this current study^17^. Since this earlier study we have acquired fresh HT-29 cells from the ATCC, which were used in this current study. We conducted a re-analysis of the original cell line in parallel with the freshly acquired cells as a positive control, which confirmed the earlier finding of unaltered MHCI regulation following RRV infection. Thus, the earlier data were accurately determined, but the MHCI phenotype of the original HT-29 cells differs from that of the HT-29 cells obtained more recently from the ATCC.

### Rotavirus NSP1 may partially restrict bystander MHCI upregulation, but MHCI downregulation in infected cells appears to be independent of NSP1

**Figure 2:**
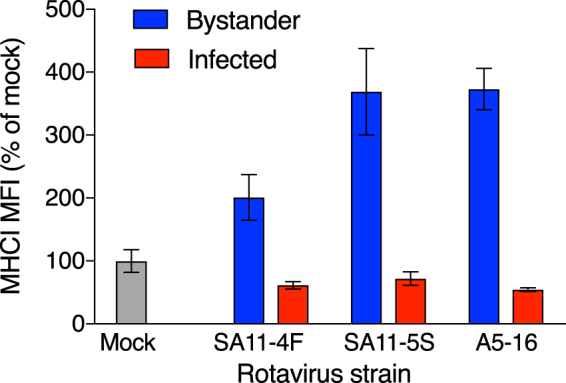
Rotavirus NSP1 reduces MHCI expression in uninfected bystander cells. Cells were mock-infected or infected with SA11-4F, SA11-5S or A5-16 rotavirus at a m.o.i. of 1. After 16 h cells were fixed and permeabilized, stained for rotavirus and total MHCI, and analysed by flow cytometry as described in the legend to Fig. 1. The mean ± S.D. of the MHCI MFI from three biological replicates is shown.

NSP1 is a major antagonist of innate immunity to rotavirus. To test a possible role for NSP1 in MHCI regulation by rotavirus, we examined the effects of HT-29 cell infection with SA11-5S and A5-16 rotaviruses, which encode defective NSP1 proteins lacking the last 17 aa or 442 aa, respectively^18,19^. SA11-4F, which encodes a functional NSP1, was included as a control^18^. As for RRV, infection with each these three rotaviruses separately at a m.o.i. of 1 induced MHCI upregulation in bystander cells over mock-infected cells (Fig. S1, Fig. 2; 0.0002 ≤ p ≤ 0.0125). Approximately 100% higher levels of total MHCI in bystander cells were observed following infection with SA11-5S and A5-16 compared to SA11-4F (Fig. S1, Fig. 2; 0.0037 ≤ p ≤ 0.02), suggesting that NSP1 partially limits bystander MHCI expression. However, as similar levels of MHCI were observed in SA11-4F-, SA11-5S- and A5-16-infected cells (Fig. 2; p > 0.05), it appears that NSP1 is not required for the MHCI downregulation in rotavirus-infected cells.

### The inhibition of MHC I expression in infected cells requires rotavirus replication, whereas bystander cell MHCI upregulation is virus replication-independent

**Figure 3:**
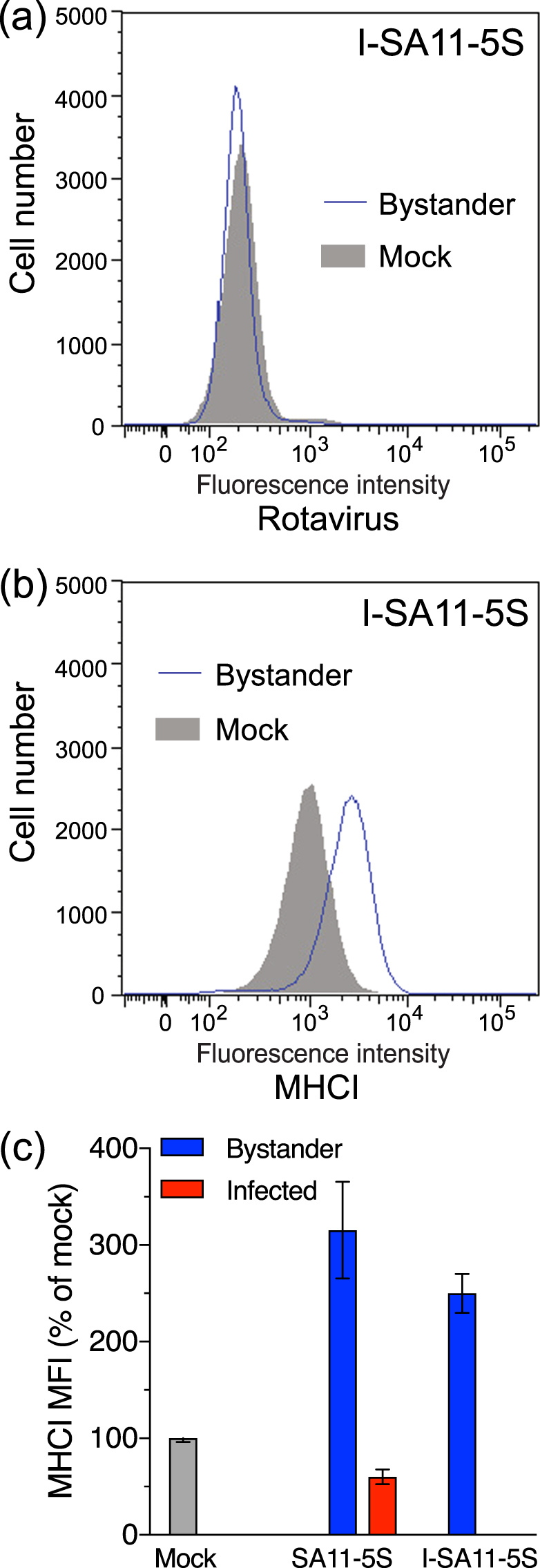
Influence of rotavirus replicative ability on total MHCI expression in infected cells and bystander uninfected cells. Cells were mock-infected, infected with SA11-5S at a m.o.i. of 1, or exposed to a corresponding inoculum of inactivated SA11-5S (I-SA11-5S). After 16 h cells were fixed and permeabilized, stained for rotavirus (**a**) and total MHCI (**b**), and analysed by flow cytometry as described in the legend to Fig. 1. (**c**) The mean ± S.D. of the MHCI MFI from three biological replicates is shown.

As SA11-5S rotavirus strongly induces MHCI expression in bystander cells, the role of rotavirus replication in regulating MHCI expression was examined using inactivated (I-) SA11-5S. As expected, rotavirus antigen was not detected in cells exposed to I-SA11-5S, as only a single cell population was present, which stained indistinguishably from mock-exposed cells (Fig. 3a). This also further confirms that our flow cytometric staining for rotavirus antigen accurately discriminates between infected and bystander cells, as we have reported previously using the same protocol^17^. MHCI levels on I-SA11-5S-exposed cells were elevated over MHCI levels on mock-infected cells (Fig. 3b,c). In these experiments, cells that were infected with SA11-5S at a m.o.i. of 1 (equivalent to the I-SA11-5S dose) showed histograms of virus antigen and MHCI staining that were almost indistinguishable from those for SA11-5S at this m.o.i. that are presented in Fig. S1, as expected. MHCI levels on the bystander cell population following infection with SA11-5S were similar to those on cells exposed to I-SA11-5S (Fig. 3c; p > 0.05). Therefore, rotavirus replication is required for the inhibition of MHCI expression, but dispensable for the increased MHCI expression in bystander cells.

### Effect of rotavirus input level on MHCI modulation

**Figure 4:**
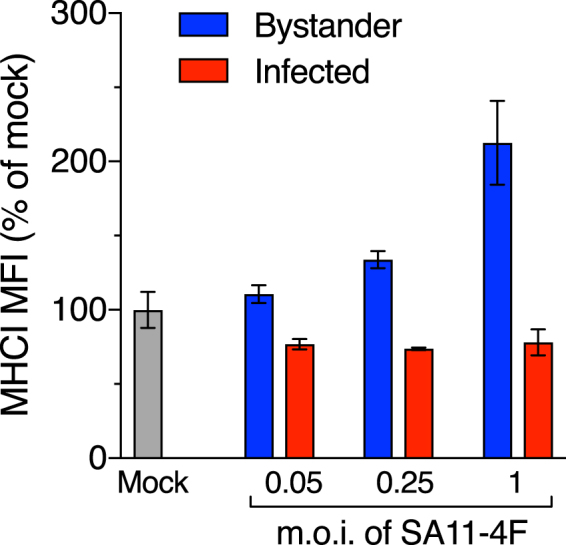
Effect of rotavirus m.o.i. on total MHCI expression in infected cells and bystander uninfected cells. Cells were mock-infected or infected with SA11-4F at m.o.i. of 0.05, 0.25 or 1 for 16 h. Cells were fixed and permeabilized, stained for rotavirus and total MHCI, and analysed by flow cytometry as described in the legend to Fig. 1. The mean ± S.D. of the MHCI MFI from three biological replicates is shown.

As expected, the proportion of cells infected by SA11-4F increased with m.o.i., being approx. 6% and 18% at m.o.i. of 0.05 and 0.25, respectively (Fig. S2). The histograms of virus antigen and MHCI staining for SA11-4F at a m.o.i. of 1 in these experiments were almost indistinguishable from those illustrated in Fig. 2, with approx. 70% of cells infected. Infection with SA11-4F at a m.o.i. of 0.05 had no effect on total MHCI levels on bystander cells (p > 0.05), whereas m.o.i. of 0.25 and 1 produced successive increases in total MHCI levels (Fig. S2, Fig. 4; 0.008 ≤ p ≤ 0.009). Infected cell expression of MHCI was reduced to a similar level at each m.o.i. tested (Fig. 4). Thus, exposure to rotavirus at m.o.i. from 0.25 to 1 progressively increases MHCI levels in bystander cells. In contrast, the extent of MHCI downregulation in rotavirus-infected cells is unaffected by m.o.i. in the range of 0.05 to 1.

### Enhancement of MHC I expression in bystander cells depends at least partially on type I IFN signalling

**Figure 5:**
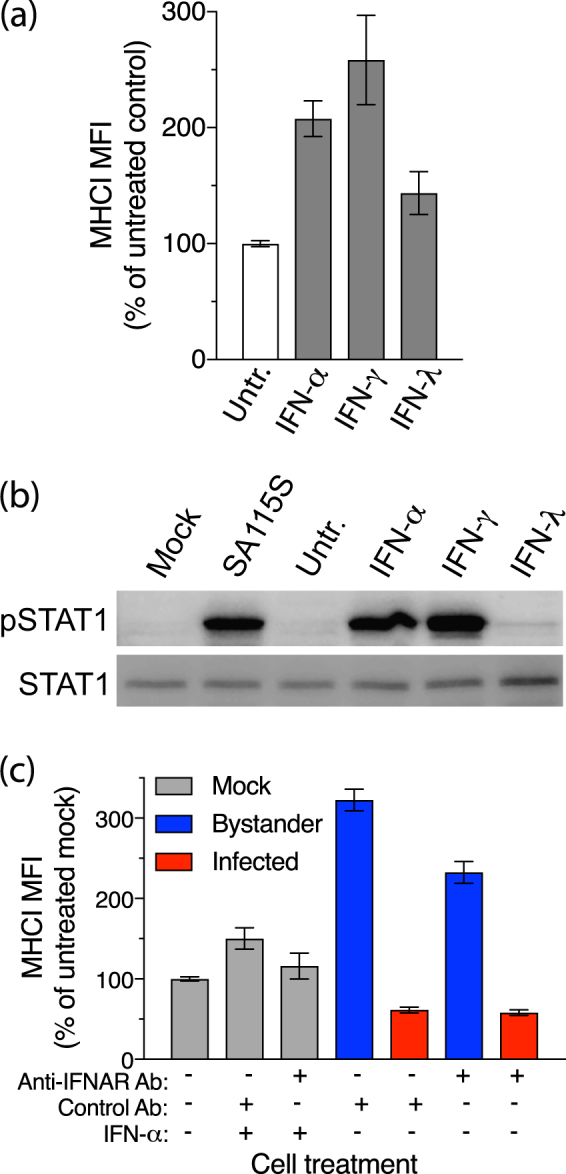
Involvement of IFN receptor signalling in modulation of MHCI expression by rotavirus. (**a**) Cells left untreated (Untr.) or treated with IFN-α, IFN-γ or IFN-λ for 16 h were fixed and stained for total MHCI and analysed by flow cytometry. (**b**) Cells were mock-infected (Mock) or infected with SA11-5S at a m.o.i of 1 for 16 h, or left untreated or treated with IFNs as for (**a**), and analysed by Western blot for levels of STAT1 or phosphorylated STAT1 (pSTAT1). Full-length blots are presented in Supplementary Figure 3. (**c**) Cells were mock-infected or infected with SA11-5S at a m.o.i. of 1 in the presence of IFN-α and/or blocking antibodies to IFNAR1, or control antibodies. After 16 h cells were fixed and permeabilized, stained for MHCI and rotavirus, and analysed by flow cytometry. For (**a**,**c**), the mean ± S.D. of the MHCI MFI from three biological replicates is shown.

The pattern of MHCI increases in bystander cells suggested that infected cells may secrete a cytokine that acts on bystander cells to induce MHCI expression. As IFNs are known to stimulate MHCI expression, we analysed the effects of IFN-α, IFN-γ, and IFN-λ on MHCI levels in HT-29 cells. All three IFN types induced MHCI expression, in the order IFN-γ > IFN-α > IFN-λ (Fig. 5a; 0.0003 ≤ p ≤ 0.0155). The ability of these IFN types to activate STAT1 was examined by Western blotting, using SA11-5S as a positve control. The expression of activated STAT1 (pSTAT1) was strongly induced by IFN-α and IFN-γ but only weakly induced by IFN-λ (Fig. 5b). It is possible that these HT-29 cells do not express high levels of the IFN-λ receptor. During rotavirus infection, HT-29 cells do not express IFN-γ^20^ and have not been reported to express IFN-λ. However, IFN-λ detection has been reported following HT-29 cell infection with vesicular stomatitis virus^21^. For these reasons, we further analysed a potential role for type I IFN in bystander cell MHCI upregulation by blocking signalling through the type I IFN receptor (IFNAR). The level of MHCI induced in mock-infected cells treated with IFN-α was reduced by 68% in the presence of optimal levels of an IFNAR2-blocking antibody, compared to an isotype-matched control antibody, demonstrating partial but significant blockade of type I IFN signalling by this anti-IFNAR2 antibody under the conditions of our experiments (Fig. 5c; p = 0.0475). The extent of the MHCI increase induced by IFN-α in this experiment, although significant (p = 0.0031), was approximately 50% less than that in the experiment shown in Fig. 5(a), possibly due in part to a slight inhibitory effect of the control antibody. In SA11-5S-exposed cell cultures, MHCI expression decreased by 40% in bystander cells treated with the IFNAR2-blocking antibody, compared with the bystander cells in cultures treated with control antibody (Fig. 5c; p = 0.0012). These results provide evidence that the MHCI induction in bystander cells is at least partially due to paracrine signalling by type I IFN secreted from infected cells. HT-29 cells are known to produce type I IFN when infected with rotavirus strains, such as RRV, that are capable of repressing IFN expression^6,22,23^. This is consistent with the proposal that type I IFN may be mainly responsible for the increased bystander cell MHCI expression observed in our experiments using RRV and SA11-4F, as well as for SA11-5S.

### Expression of genes involved in MHC I regulation is elevated in bystander cells but not rotavirus-infected cells

**Figure 6:**
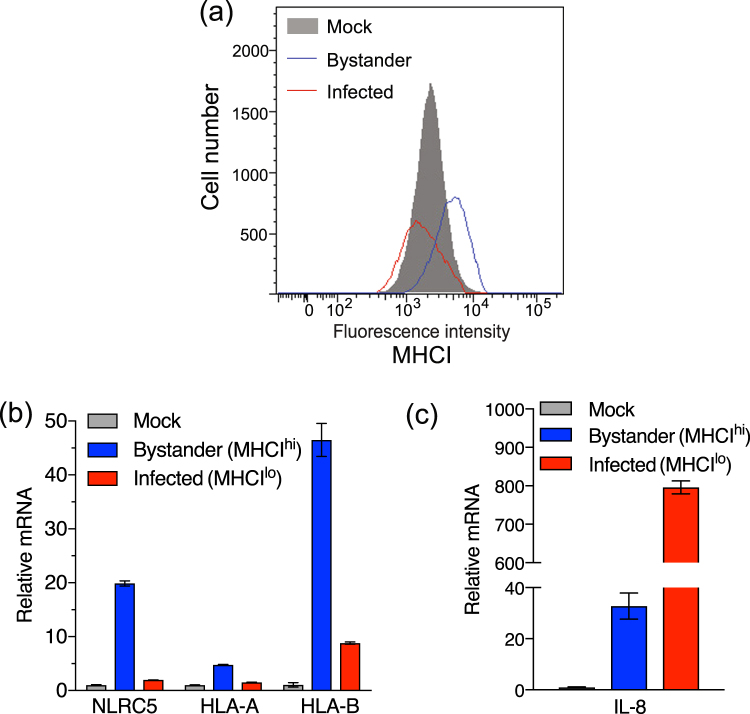
Transcriptional regulation of MHCI in rotavirus-infected and bystander cells. HT-29 cells were mock-infected or infected with RRV at a m.o.i. of 0.5 for 16 h, fixed with methanol and stained for rotavirus antigen and MHCI. Infected (MHCI^lo^) and uninfected (MHCIhi) cell populations in the RRV-infected cell cultures were collected by cell sorting (**a**). Total cellular RNA was extracted from the sorted cell populations and mock-infected cells. Relative levels of mRNA were determined by qPCR for HLA-A, HLA-B and NLRC5 (**b**), and IL-8 (**c**). The mean ± S.D. of triplicate analyses is shown.

To evaluate if MHCI and genes involved in its expression are transcriptionally regulated in rotavirus-infected and bystander cells, rotavirus-exposed cells were stained, fixed using methanol and sorted into MHCI^hi^/rotavirus^neg^ (bystander) and MHCI^lo^/rotavirus^pos^ (infected) populations. Unsorted cells from a mock-infected culture were included for comparison (Fig. 6a). Analysis by qPCR of extracted RNA was used to determine the relative mRNA levels of NLRC5, HLA-A, HLA-B, IFN-β and IL-8. Compared to levels in mock-infected cells, NLRC5 mRNA was clearly induced in bystander cells, but not infected cells. HLA-A expression was slightly induced in bystander but not infected cells, while HLA-B was robustly induced in bystander cells, with a small increase also observed in infected cells (Fig. 6b). We could not reliably measure the increases in IFN-β mRNA levels in infected or bystander cells relative to mock-infected cells, due to undetectably low levels in the mock-infected samples. However, based on the differences between the Ct values for the infected and mock-infected samples and the last cycle of the qPCR program, IFN-β mRNA levels were increased over mock-infected cells by >65-fold in infected cells and >15-fold in bystander cells. This demonstrated that IFN-β was induced in response to rotavirus infection, with the great majority in the infected cells. IFN-β was detected in RRV-infected HT-29 cells in previous studies by our group and others, as either secreted protein or elevated mRNA level^13,24^. As expected from earlier work^25^, IL-8 was strongly induced in infected cells, but not bystander cells (Fig. 6c). These data show that expression of MHCI genes, and the gene encoding the MHCI master regulator NLRC5, is induced in bystander cells but restricted in rotavirus-infected cells. In addition, IFN-β and IL-8 are expressed primarily in infected cells.

### Nuclear accumulation of activated STAT1 and expression of IRF1 is blocked in rotavirus-infected cells but not bystander cells

**Figure 7:**
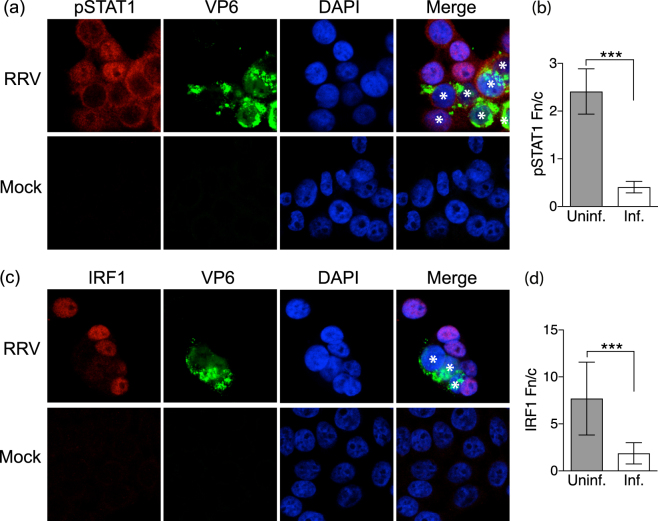
Localization of activated STAT1 and IRF1 in rotavirus-infected and bystander cells. HT-29 cells were infected with RRV at a m.o.i. of 1 or mock-infected. After 7 h cells were fixed, permeabilised and stained with antibodies to rotavirus VP6 and phosphorylated STAT1 (pSTAT1; **a**,**b**) or VP6 and IRF1 (**c**,**d**). Nuclei were stained with DAPI and representative images obtained by confocal microscopy. The VP6-positive (infected) cells in the merged images are indicated with white asterisks. Nuclear to cytoplasmic fluorescence ratios (Fn/c) of pSTAT1 (**b**) and IRF1 (**d**) were calculated for the uninfected and infected cell populations, analysing 7 to 14 randomly selected cells of each population type from 2 independent experiments. Images were obtained at ×1000 magnification. ***P < 0.0001.

Activated STAT1 has an important role in regulating MHCI expression. The expression of activated STAT1 was investigated in rotavirus- and mock-infected HT-29 cell cultures. At 8 h p.i. and a m.o.i. of 1, both infected and uninfected cells were clearly evident (Fig. 7a). Compared to mock-infected cells, which showed little pSTAT1 expression, both infected and uninfected (bystander) cells showed substantial pSTAT1 expression (Fig. 6a). However, pSTAT1 was primarily located in the cytoplasm of infected cells and in the nucleus of uninfected cells (Fig. 7b). This supports the hypothesis that IFNα/β is secreted from rotavirus-infected cells and acts in both a paracrine and autocrine manner to activate STAT1, but STAT1 nuclear translocation is inhibited in the infected cells. The expression of IRF1, which can be stimulated by STAT1 and play a role in MHCI expression, was also examined (Fig. 7c). IRF1 was present in the nucleus of uninfected (bystander) cells but largely absent from rotavirus- and mock-infected cells (Fig. 7d). This is consistent with the possibility that IFNα/β induction of IRF1 expression may play a role in the MHCI upregulation induced in bystander cells.

## Discussion

The presentation of peptides via MHCI to CTL is crucial for the adaptive immune response to viral infections. Here we use an epithelial cell culture model to show that rotavirus prevents MHCI upregulation in infected cells. Our data suggest that this is at least partially type I IFN-dependent. It is probably due to the inhibition of STAT1 nuclear translocation and subsequent expression of NRLC5, the master activator of MHCI expression. Furthermore, baseline MHCI protein levels are reduced in rotavirus-infected cells. Additional studies that include rotavirus-infected hosts and immune cell analyses are needed to confirm the findings from our cell culture model. With this caveat, when taken together, these cell culture data suggest that rotavirus might have the capacity to reduce the exposure of infected cells to killing by CTL.

Several lines of evidence presented here also support a role for type I IFN secreted from rotavirus-infected cells in the induction of elevated MHCI expression in bystander cells that show no evidence of rotavirus replication. Firstly, infection with SA11-5S, which cannot degrade IRF3/7 and thus inhibit IFN-β expression due to a defective NSP1, induced higher bystander cell MHCI expression than infection with SA11-4F, which does degrade IRF3/7. Secondly, MHCI upregulation in bystander cells was at least partially inhibited by IFNAR-blocking antibodies. Thirdly, in the rotavirus-infected cell cultures, activated STAT1 was observed in the nucleus of bystander but not infected cells. Lastly, IFN-β mRNA was induced in response to rotavirus infection, mostly in the infected cells. In response to rotavirus infection, the release of IFN from immune cells, such as dendritic cells, may be a major contributor to IFN expression *in vivo* ^5,26,27^. If this is the case, then signalling through STAT1 following stimulation by IFN produced in dendritic cells should also be blocked in infected enterocytes, limiting MHCI expression. It is possible that other cytokines, such as IFN-λ, also may contribute to bystander MHCI regulation in infected HT-29 cells.

The extent of the MHCI upregulation on bystander cells may be considered as an indication of the level of MHCI that might be induced in cells infected by rotavirus, should this virus lack the ability to inhibit MHCI expression. This MHCI upregulation on bystander cells is of great potential importance in understanding how rotavirus infection impacts on immune regulation and immune-mediated diseases, such as type 1 diabetes. Rotavirus infection is associated with progression to type 1 diabetes in children, and accelerates type 1 diabetes development in diabetes-prone NOD mice^28^. RRV rotavirus infection of NOD mice leads to MHCI upregulation on plasmacytoid dendritic cells and B cells in gut- and pancreas-draining lymph nodes^29,30^. However, <2% of these cells show evidence of RRV infection, indicating that they are predominantly bystander cells^31^. MHCI also is upregulated on the insulin-producing pancreatic β cells in RRV-infected NOD mice, without evidence of pancreatic RRV infection^32^. This bystander activation, and the rotavirus acceleration of type 1 diabetes, depend on type 1 IFN production by plasmacytoid dendritic cells and lymphocytes, and on type 1 IFN signaling^5,29^. Furthermore, the identification here of rotavirus upregulation of MHCI on bystander human intestinal epithelial cells also will assist in understanding the role of this virus in intestinal immune disregulation and related disorders, such as celiac disease^33^.

We found that mRNA encoding HLA-A was stimulated at a lower level than mRNA for HLA-B in bystander cells. This may reflect cell-specific factors or the different promoter activities of these genes^34^. Regardless of this, induction of both genes was strongly suppressed in infected cells. We also observed a lack of IRF1 protein expression in infected, but not bystander, cells. IRF1 can be induced by IFN-α/β or IFN-γ activation of STAT1, and is constitutively translocated to the nucleus where it can bind sites in MHCI gene promoters. Therefore, the absence of IRF1 expression in infected cells may also play a role in the observed lack of MHCI upregulation.

A number of viruses, most notably herpesviruses, have been found to actively interfere with peptide presentation on MHCI^35^. Various mechanisms of viral inhibition of this peptide presentation have been identified, including inhibition of peptide translocation to the ER through interference with TAP, interference with peptide loading onto MHCI, retention of MHCI in the ER and redirection of MHCI away from normal trafficking to the cell surface. The mechanism leading to the observed reduction in MHCI levels in rotavirus-infected cells is not known. We observed that total and cell surface MHCI levels are reduced during infection, which does not rule out the degradation of MHCI, either in a targeted way or by redirection of MHCI to a degradative pathway through inhibition of correct loading complex formation. Rotavirus NSP1 causes proteasome-dependent degradation of host cell targets including IRFs and NF-κB^9,10^. However, NSP1 does not appear to be involved in rotavirus-induced MHCI downregulation, as the NSP1-defective viruses SA11-5S and A5-16 still showed this effect. It is possible that rotavirus NSP3 may contribute to MHCI downregulation through inhibition of translation^36^.

Antibody and T cell responses to rotavirus are important in viral clearance and protection against reinfection^37^. No information has been reported on the ability of rotavirus to evade adaptive immune responses. Studies to determine the relevance of our findings to rotavirus infection in an animal host are needed. The hypothesis that the importance of T cells in rotavirus clearance might be diminished by the ability of rotavirus to limit MHCI expression in infected cells could then be tested. While MHCI is still expressed on infected cells in this study, MHCI levels are 4- to 5-fold lower than those on bystander cells, and approximately 40% lower than those on mock-infected cells. In a rotavirus-infected host, it is possible that this could lead to lower levels of peptide presentation to CTL, potentially allowing greater survival of infected cells. However, the absence of cell surface MHCI can lead to detection and killing by natural killer (NK) cells^38^. Possibly, the partial downregulation of MHCI seen in infected cells relative to normal levels might occur *in vivo*, and still provide sufficient MHCI to prevent detection by NK cells. These hypotheses remain to be tested.

These studies provide insights into the process of MHC I regulation in intestinal cells, including the roles of IFN-α/β, NLRC5 and IRF1. Overall, it is shown here that rotavirus inhibition of STAT1 signaling also is associated with reduced MHCI expression in infected cells. This supports the proposal that rotavirus inhibition of STAT1 signaling also reduces the expression of MHCI in infected cells. It is conceiveable that this may also occur during rotavirus infection *in vivo*, which might impact on cellular immunity to rotavirus.

## Methods

### Cells, viruses and infection

HT-29 cells were obtained directly from the ATCC and maintained as described previously^25^. Cells used had been passaged <30 times. SA11-4F and SA11-5S rotaviruses were obtained from John Patton^18^. A5-16 rotavirus was provided by Koki Taniguchi^19^. Rotaviruses were propagated in MA104 cells and titres determined by infectivity titration as before^39^. Rhesus monkey rotavirus strain RRV, and simian rotavirus strains SA11-4F and SA11-5S, were purified using glycerol gradient ultracentrifugation as previously described^25^. Bovine rotavirus strain A5-16 was used in a non-purified form from clarified cell culture lysate^40^. For infection, cells were incubated with rotavirus inoculum in serum-free medium for 1 h, after which the inoculum was removed and replaced with medium containing 10% fetal bovine serum for the remainder of the indicated infection period. All viruses were activated with porcine trypsin (10 μg ml^−1^, Sigma) for 20 min at 37 °C before infection.

### Flow cytometry and cell sorting

HT-29 cells were mock-infected or infected with rotavirus at the indicated m.o.i. for 17 h. These m.o.i. were determined in HT-29 cells for the growth of MA104 cell-propagated rotaviruses. For analysis of cell surface MHCI, cells in monolayers were placed in suspension by brief treatment with trypsin-EDTA and stained with APC-conjugated mouse anti-HLA-ABC (clone W6/32) or isotype control antibody (clone eBM2a) from eBioscience^17^, followed by fixation and permeabilization with the BD Biosciences Cytofix/Cytoperm kit according to the manufacturer’s instructions. After blocking with 10% goat serum, rotavirus-infected cells were stained with rabbit anti-rotavirus antiserum or control polyclonal antibodies diluted 1 in 500 and Pacific Blue-conjugated goat anti-rabbit IgG (Life Technologies), as before^17^. Stained single cells were quantified using a LSRII Flow Cytometer (BD) and analysed with FlowJo software (Tree Star Inc). For analysis of total MHCI levels, cells were detached, fixed, permeabilised, and blocked as above prior to staining for MHCI and rotavirus as above. The mock-infected, bystander and rotavirus-infected cell populations showed indistinguishable levels of staining with control antibodies (polyclonal or clone eBM2a).

For IFN stimulation of MHCI, HT-29 cells were left untreated or treated with IFN-α (1000 U ml^−1^, PBL Interferon Source), IFN-γ (50 ng ml^−1^, BD) or IFN-λ (IL-29, 50 ng ml^−1^, Cell Signaling) for 16 h. For IFN-α/β receptor (IFNAR2) blocking, cells were treated with the optimal level of MMHAR-2 antibody (10 μg ml^−1^, PBL Interferon Source) or a matched control antibody (UPC10, 10 μg ml^−1^, Sigma) throughout the 16 h period of IFN-α treatment or infection, followed by staining and flow cytometry for total MHCI and rotavirus as above. The optimal level of MMHAR-2 antibody we determined was twice the manufacturer’s recommended maximum of 5 ug/ml. This antibody has been successfully used by others at 5 ug ml^−1^ to inhibit HIV-1 and R848-induced PDL-1 expression on human neutrophils^41^ and has been useful *in vivo*, to block IFN-stimulated gene expression in mice^42^.

For cell sorting experiments, mock-infected or rotavirus-infected cells were fixed with ice cold methanol for 10 min, washed with wash buffer consisting of PBS containing DTT (1 mM) and RNasin (1000 U ml^−1^, Promega), blocked with 10% goat serum in wash buffer, and stained with antibodies to MHCI and rotavirus, diluted in wash buffer. Mock cells were left unsorted and MHCI^hi^/rotavirus^neg^ and MHCI^lo^/rotavirus^pos^ populations collected from the rotavirus-infected cell cultures using a FacsAria cell sorter (BD). RNA was extracted using the RNeasy mini kit with DNAse treatment (Qiagen).

### Western Blotting

HT-29 cells were left untreated or treated with IFN-α, IFN-γ or IFN-λ for 16 h. Alternatively, cells were mock-infected or infected with SA11-5S at an m.o.i of 1. These processes were performed as for the flow cytometry experiments. Cell lysates were analysed by Western blotting for total STAT1 and phosphorylated STAT1 (pSTAT), as previously described^15^.

### Quantitative real-time PCR (qPCR)

Reverse transcription was performed using the Tetro cDNA synthesis kit (Bioline) using random primers. qPCR was carried out using the SensiFAST SYBR Lo-ROX kit (Bioline) or the Brilliant II Probe master Mix (Agilent) and the MX3005P qPCR system (Agilent). Primers used: 18s rRNA forward 5′-CGGCTACCACATCCAAGGAA-3′, 18s rRNA reverse 5′-GCTGGAATTACCGCGGCT-3′; NLRC5 forward 5′-CTGGCCAGTCTCACCGCACAA-3′, NLRC5 reverse 5′-CCAGGGGACAGCCATCAAAATC-3′; HLA-A forward 5′-AAAAGGAGGGAGTTACACTCAGG-3′, HLA-A reverse 5′-GCTGTGAGGGACACATCAGAG-3′; HLA-B forward 5′-CTACCCTGCGGAGATCA-3′, HLA-B reverse 5′-ACAGCCAGGCCAGCAACA-3′; IL-8 forward 5′-TCTGCAGCTCTGTGTGAAGG-3′, IL-8 reverse 5′-AGTGTGGTCCACTCTCAATC-3′. Probe/primers used: Human IFNB1 taqman primer/probe FAM-MGB (Hs01077958_s1, ThermoFisher Scientific). Data were analysed using MxPro software (Agilent) and the ΔCt method, employing 18s rRNA as the reference gene.

### Microscopy

HT-29 cells on glass coverslips were mock-infected or infected with RRV for the indicated times. Cells fixed with 1:1 methanol/acetone were incubated with mouse monoclonal antibody RVA to rotavirus VP6^43^, and rabbit monoclonal antibodies to phospho-STAT1 (Tyr701) or IRF1 (Cell Signaling). Cells were stained with anti-mouse IgG Alexa Fluor 488-conjugated or anti-rabbit IgG Alexa Fluor 594-conjugated secondary antibodies (Life Technologies). Coverslips were mounted onto slides using Prolong Gold Antifade reagent with DAPI (Life Technologies). Images were obtained with an LSM700 confocal microscope at ×1000 magnification (Zeiss). Fluorescence levels in the nucleus (Fn) and the cytoplasm (Fc), minus background fluorescence, were determined using Image J version 1.47 software^44^ and used to calculate the nuclear to cytoplasmic ratio (Fn/c) of phospho (p)-STAT1 and IRF1 in infected and uninfected cells. From 7 to 14 randomly selected cells from 2 independent experiments were analysed.

### Statistical analysis

Student’s *t*-test was used, including Welch’s correction as appropriate. In Figures, bars represent the mean and S. D.

### Data availability

No datasets were generated or analysed during the current study.

## Electronic supplementary material

Supplementary Information
