# Supplementary material for: MHC class I expression in intestinal cells is reduced by rotavirus infection and increased in bystander cells lacking rotavirus antigen
Source: Sci Rep. 2018 Jan 8;8:67. doi: 10.1038/s41598-017-18464-x (PMC5758578; doi:10.1038/s41598-017-18464-x)
Supplement: Supplementary file 1 — Supplementary Information [file 41598_2017_18464_MOESM1_ESM.pdf]

MHC class I expression in intestinal cells is reduced by rotavirus infection and increased in bystander cells lacking rotavirus antigen

Supplementary Figures

Gavan Holloway, Fiona E. Fleming and Barbara S. Coulson

Department of Microbiology and Immunology, The University of Melbourne at the Peter Doherty Institute for Infection and Immunity, Melbourne, Victoria, Australia

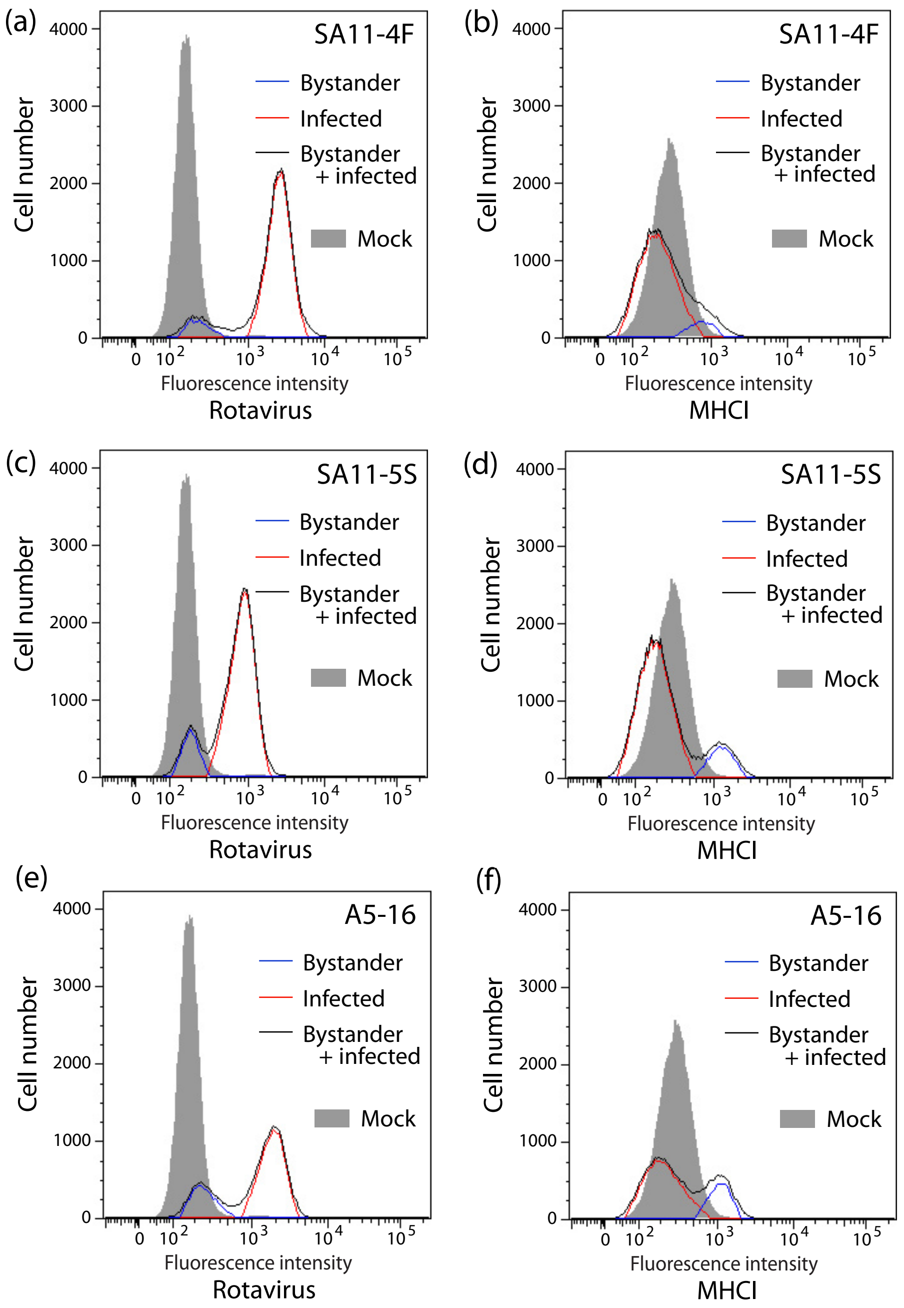

Supplementary Figure S1. Flow cytometric histograms of cell populations in rotavirus- or mock-infected HT-29 cell cultures. Cells were mock-infected or infected with SA11-4F (a, b), SA11-5S (c, d) or A5-16 rotavirus (e, f) at a m.o.i. of 1. After 16 h cells were fixed and permeabilized, stained for rotavirus (a, c, e) and total MHCI (b, d, f), and analysed by flow cytometry as described in the Legend to Fig. 1.

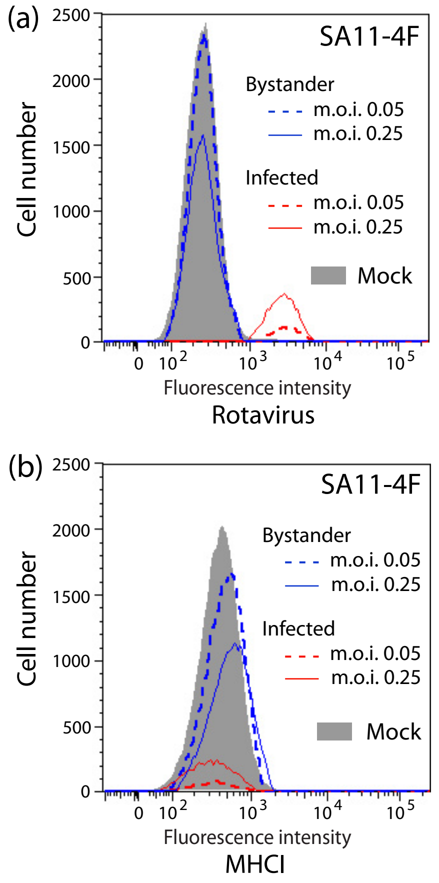

Supplementary Figure S2. Flow cytometric histograms of cell populations in HT-29 cell cultures infected with SA11-4F at a range of m.o.i. Cells were mock-infected or infected at m.o.i. of 0.05, 0.25 or 1. After 16 h cells were fixed and permeabilized, stained for rotavirus (a) and total MHCI (b), and analysed by flow cytometry as described in the Legend to Fig. 1.

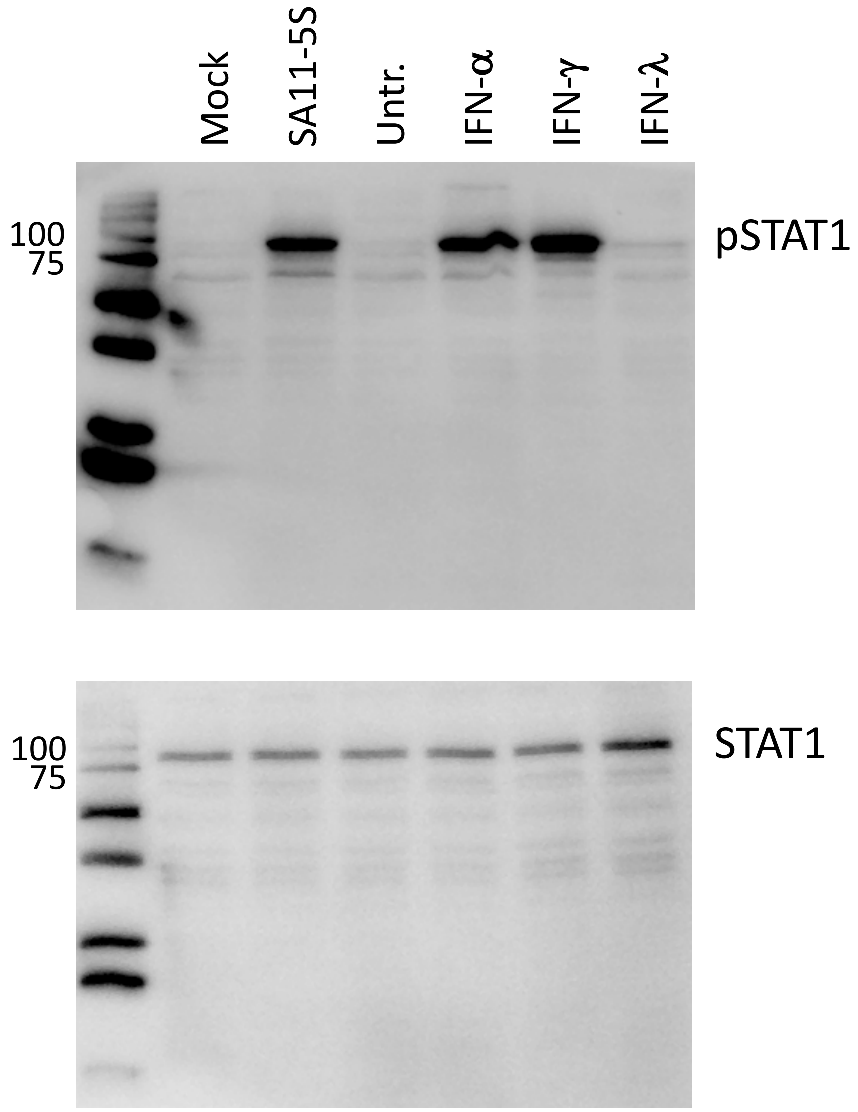

Supplementary Figure S3. Full-length Western blots corresponding to the cropped STAT1 blots shown in Fig. 5(b). Cells were mock-infected (Mock) or infected with SA11-5S at a m.o.i of 1 for 16 h, left untreated (Untr.) or treated with IFN-α, IFN-γ or IFN-λ for 16 h, and analysed by Western blot for levels of STAT1 or phosphorylated STAT1 (pSTAT1). Molecular size markers were included in the left lane of each gel blot, and are labelled in kDa.
